# Dynamics of energy transport on hydromagnetic Casson slippery nanoflow over curved surface

**DOI:** 10.1016/j.heliyon.2024.e30638

**Published:** 2024-05-03

**Authors:** Bhargavi N, Poornima T

**Affiliations:** Department of Mathematics, School of Advanced Sciences, Vellore Institute of Technology, Vellore, Tamilnadu, 632014, India

**Keywords:** Curved stretching sheet, Trihybrid nanofluid, Casson fluid, Non-linear thermal radiation, Magnetic field

## Abstract

The study of fluid flow over curved surfaces is crucial in various engineering applications, such as designing aircraft wings, turbines, and submarines. Curved surfaces are being explored in various biomedical applications, such as designing stents for blood vessels and implants for bones and joints. Concerning the present applications of curved stretching sheets on fluid dynamics along with trihybrid nanofluids, this study is unique and fills the research gaps and offers solutions to several issues. This work looks at the flow through the boundary layer of an electrically conductive trihybrid nanofluid and the convection heat exchange of a Casson fluid across a curved stretched surface encircled within a circle of radius *R*. The study considers the effects of thermal radiation using the non-linearized Rosseland approximation, as well as a magnetic field, and hydromagnetic slip. The flow as well as the transfer of heat problem is mathematically described by curvilinear coordinates. Using the combination of the shooting technique and the Runge-Kutta method, similar solutions to the modeled partial differential equations are produced, and the set of non-linear ordinary differential equations with a boundary value solver is implemented through the MATLAB program. The study finds the influence of several limits on critical characteristics such as fluid velocity, coefficient of skin friction, pressure, temperature, and rate of heat exchange over a surface. The findings are shown in tables and graphs. Additionally, a comparative analysis between the current findings and those found in the literature is provided. Blood-containing nanoparticles (*GO-SWCNTs-MWCNTs*) on curved surfaces could improve drug delivery effectiveness, growth of synthetic tissues or organs with complex structures and effective cancer therapy treatment.

## Graphical abstract

**Figure:**
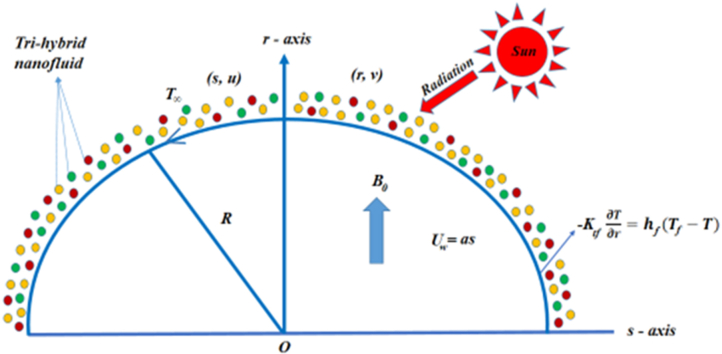

**Table:** NOMENCLATURE

Dimensionless parameters		Dimensional parameters	
*P*	Pressure	*μ*	Dynamic viscosity(*Pa.s*)
*M*	Magnetic parameter	*α*	Thermal diffusivity (*m*^*2*^*.s*^*−*^*^1^*)
*Rd*	Radiation parameter	*ρ*	Intensity (*kg.m*^*−*^*^3^*)
*θ*_*w*_	Temperature ratio parameter	*U*_*w*_	Stretching velocity in s-direction (*m.s*^*−*^*^1^*)
*L*_*1*_	Slip length	*σ*	Electrical conductivity (*m.s*^*−*^*^1^*)
*K*	Radius of curvature	*ν*	Viscosity in motion (*m*^*2*^*.s*^*−*^*^1^*)
*Bi*	Biot number	*p*	Pressure (*Pa*)
$\beta_{1}$	Casson parameter	$\beta_{f}$	Thermal expansion (*K*^*−*^*^1^*)
*η*	Dimensionless variable	τ_w_	shear tension at the wall (*N.m*^*−*^*^2^*)
*f*	Stream function	*k*	Temperature conductivity (*W.m*^*−*^*^1^.K*^*−*^*^1^*)
*θ*	Thermal function	*C*_*p*_	Specific heat(*J.kg*^*−*^*^1^.K*)
*φ*	Concentration function	*q*_*w*_	Heat flux(*W.m*^*−*^*^2^*)
*Pr*	Prandtl's number	*v, u*	Velocity components in vertical and horizontal directions (*m.s*^*−*^*^1^)*
Re_*s*_	Local Reynold's number	*s,r*	Curvilinear Coordinates (*m)*
*Nu*_*s*_	Local Nusselt number	$T_{\infty }$	Ambient Temperature (*K*)
Subscripts		$T_{w}$	Sheet Temperature(*K*)
*f*	Base fluid	T	Temperature(*K*)
*nf*	Nanofluid	*R*	The radius of curvature (*m*)
*hf*	Hybrid nanofluid	K*	Mean absorption coefficient (−)
*tf*	Trihybrid nanofluid	σ*	Stefan–Boltzmann constant (−)

## 1 Introduction

Nanofluids surpass normal fluids in terms of thermal efficiency and stability. These fluids provide effective heating and cooling systems for commercial uses such as automotive, electronics, solar, and manufacturing. Nanofluids are formed when particles and base materials collide, usually with diameters from 1 to 100 nm. Metallic nanoparticles (e.g. gold, copper, and silver) and non-metallic nanoparticles (e.g. graphene, carbon nanotubes, and graphene oxide). They may dissolve in many fluids, like oil, blood, ethylene glycol, and water. Ultimately, nanoliquids have exciting opportunities for enhancing heat transfer efficiency, but they also confront several obstacles, including long-lasting stability and compatibility with materials concerns. For example, hybrid nanofluids are developed to improve design, dependability, and affordability for a variety of real-world uses. Nanofluids that are hybrid, combine the properties of two different nanoparticles in a base fluid. The goal behind a nanofluids that are hybrid is to improve its thermal conductivity, transfer of heat efficiency, electrical conductivity, and fluid stability as compared to a single nanofluid. Hybrid nanofluids have a wide range of uses, including welding, lubrication, automated reactors, electronics cooling, and biomedical engineering. Several studies were undertaken to indicate a significant increase in the rate of heat exchange in nanofluids and hybrid nanofluids under diverse conditions. All these inspired the researchers to create more efficient nanofluids with higher heat transfer and thermal conductivity. This culminated in the development of a new working fluid, the Trihybrid nanofluid concept. Trihybrid nanofluid is a mixture of three different varieties of tiny particles and base liquids. Each form of nanoparticle has unique features that may enhance heat transfer. Abbasi et al. [1], studied the thermal performance of a (*Al*_*2*_*O*_*3*_*–SiO*_*2*_*–TiO*_*2*_*/C*_*2*_*H*_*6*_*O*_*2*_) modified hybrid nanofluid model with a curved radiated surface. Fatima et al. [2], analyzed Cattaneo-Christov heat transfer via non-linear radiation ternary, hybrid, and single mass diffusion on a stretched surface. Maranna et al.*,* [3] studied the movement of a viscous ternary nanoliquid across a decreasingly permeable media, including sources, sinks, and radiation of heat. Thakur and Sood [4] studied trihybrid nanofluid movement over a Riga plate stretched and warmed having a different thicknesses.

The significant role of liquid movement around a curved stretch sheet is crucial in various industrial, technological, and engineering domains. Its wide-ranging applications encompass paper manufacturing, the aerospace industry, polymer extrusion, automotive designs, architectural designs, and the medical field. Numerous researchers [[5], [6], [7], [8], [9]] investigated the dynamics of liquid move over a curved extending surface. Kumar et al. [10], investigated the study of heat exchange via convection and the KKL associations for modeling of nanofluid movement across a curved stretched sheet. Shinwari et al. [11], performed a numerical investigation on trihybrid tiny materials flowing in convectively heated and curved sheets. Ashraf et al. [12], studied an innovative design for a double-pipe heat exchanger with arrow-shaped expanded surfaces that optimizes thermodynamics in laminar and fully developed flow.

Magnetohydrodynamics is a subject of physics that investigates the way electrically conducting liquids behave when exposed to magnetic fields, such as liquid metals, plasmas, and sodium chloride water. It combines magnetism and fluid dynamics concepts to illustrate how fluid motion interacts with a magnetic field. Refs. [[13], [14], [15], [16], [17], [18]] provides the results of several studies on MHD investigation. Prasad et al. [19], conducted a numerical investigation on non-Darcy MHD natural convection flow of Joule-heating and dissipation of viscous liquid effects from a horizontal cylinder a porous material that has internal heat production. Raju and Sandeep [20] examined the impact of Soret and Dufour on the magnetohydrodynamic motion of Casson liquid by a revolving cone containing gyrotactic bacteria. Waini et al. [21], investigated the heat exchange and MHD flow of a hybrid nanoliquid going through a porous stretching/shrinking wedge. Bhargavi et al. [22], examined the conjugate heat exchange of magnetohydrodynamics through a vertical permeable plate in a viscous liquid. Hussain et al. [23], utilized the Keller box approach to analyze heat transfer in the MHD flow of hybrid nanofluid between two vertically moving surfaces. Maheswari et al. [24], examined the MHD Forchheimer flow of *Fe*_*3*_*O*_*4*_ – *H*_*2*_*O, Cu – H*_*2*_*O*, and *Ag – H*_*2*_*O* nanofluids across a permeable stretched sheet with radiation was investigated numerically.

The current work intends to conduct research on the impacts of MHD, non-linearized thermal radiation, and convective boundary conditions on a Casson (*GO-SWCNTs-MWCNTs/Blood*) trihybrid nanofluid across a curved stretched surface with hydromagnetic slip. Numerical solutions for fluid velocity, coefficient of skin friction, pressure, temperature, and the rate of heat exchange over the surface are achieved by combining the shooting approach with the R–K technique. The findings are displayed as graphs and tables. Additionally, a comparison between the current results and those found in the literature is provided.

Concerning the present biomedical applications of trihybrid nanofluids, this study is unique and fills the gaps in research and offers solutions to several issues.

• MHD trihybrid nanofluid flow is taken into consideration, involving non-linear thermal radiation, hydromagnetic slip, and convective conditions.
• The fluid flow is on a stretching curved surface.
• The Casson fluid model is employed to investigate blood-base fluid's thinned and thickened by shear rheological aspects.
• To analyze the thermal uses of graphene oxide and carbon nanotubes, including single and multi-wall nanotubes suspended in blood-base fluid.
• Shooting with Runge-Kutta technique is used in the solution development process.
• The physical consequences of several random factors are established by graphic analysis.
• To see interesting quantities, a numerical analysis is performed and shown in tabular form.

## 2 Modeling

**Fig. (1):**
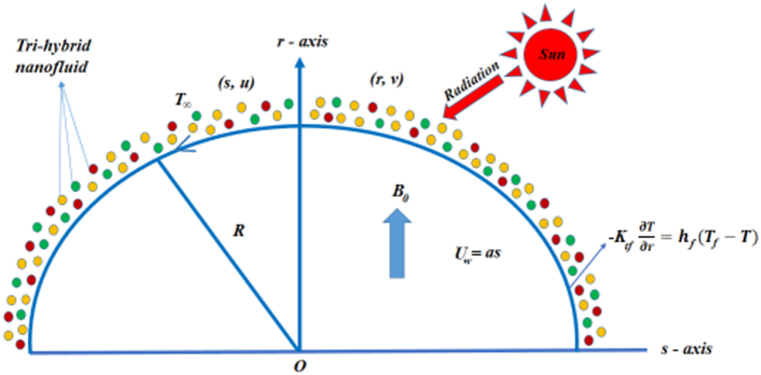
Flow diagram.

Consider a 2-D steady incompressible flow of a Casson trihybrid nanofluid on a curved stretched sheet covered in a circle with radius *R*, as depicted in Fig. (1). The sheet stretches in the *s*-direction having velocity *U*_*w*_ = *as*, here ‘*a’* is constant. A stable magnetic field of magnitude *B*_*0*_ is imposed in the *r*-direction. Choose an extremely low magnetic Reynolds number to neglect the impact of the magnetic field that is induced. Maintaining a constant temperature *T*_*w*_ for the sheet, where *T*_*w*_ > *T*_*∞*_ and *T*_*∞*_ is the temperature of the ambient liquid.

The following are the flow assumptions used in the current analysis:

❖ Curved stretching sheet. The flow is due to stretching and linear pressure
❖ Two-dimensional incompressible steady flow
❖ MHD trihybrid nanofluid flow
❖ Convective heat transfer Casson fluid model

The rheological equation of the Casson fluid for an isotropic and incompressible flow is mentioned by Refs. [25,26]:

$$\tau_{ij}=\left\{ \begin{array}{c} 2\left(p_{r}/\sqrt{2\pi }+\mu_{B}\right)e_{ij},\pi \geq \pi_{c},\\ 2\left(p_{r}/\sqrt{2\pi_{c}}+\mu_{B}\right)e_{ij},\pi <\pi_{c}, \end{array}\right.$$

In this case, $\pi =e_{ij}e_{ij}$, where $e_{ij}$ denotes for the (i,j)th components of the rate of deformation. According to the non-Newtonian model, $\pi_{c}$ represents a crucial value of this product. Furthermore, $p_{r}$ denotes the fluid's yield stress, while $\mu_{B}$ stands for the non-Newtonian fluid's dynamic viscosity. The flow and heat transfer equations under these assumptions take into account the boundary layer approximation and incorporate non-linearized thermal radiation while excluding the effects of viscous dissipation [6,27]

(1) $$\frac{\partial \left\{v\left(R+r\right)\right\}}{\partial r}+R\frac{\partial u}{\partial s}=0\text{,}$$

(2) $$\frac{u^{2}}{R+r}=\frac{1}{\rho_{tf}}\frac{\partial p}{\partial r}\text{,}$$

(3) $$v\frac{\partial u}{\partial r}+\frac{uv}{R+r}+\frac{Ru}{R+r}\frac{\partial u}{\partial s}=-\frac{R}{\rho_{tf}\left(R+r\right)}\frac{\partial p}{\partial s}+\nu_{tf}\left(\frac{1}{\beta_{1}}+1\right)\left(\frac{\partial^{2}u}{\partial r^{2}}+\frac{1}{R+r}\frac{\partial u}{\partial r}-\frac{u}{{\left(R+r\right)}^{2}}\right)-\frac{\sigma B_{0}^{2}}{\rho_{tf}}u\text{,}$$

(4) $$v\frac{\partial T}{\partial r}+\frac{Ru}{R+r}\frac{\partial T}{\partial s}=\alpha_{tf}\left(\frac{1}{R+r}\frac{\partial T}{\partial r}+\frac{\partial^{2}T}{\partial r^{2}}\right)-\frac{\frac{\partial }{\partial r}\left[\left(R+r\right)q_{r}\right]}{{\left(\rho C_{p}\right)}_{tf}\left(R+r\right)}\text{,}$$

The following are the boundary conditions that correspond to the flow and heat transfer problem [6,28]:

(5) $$\begin{array}{c} At\;r=0,u=as+L\left(\frac{\partial u}{\partial r}-\frac{u}{R+r}\right),v=0,-k_{tf}\frac{\partial T}{\partial r}=h_{f}\left(T_{f}-T\right)\text{,}\\ As\;r\to \infty ,u\to 0,\frac{\partial u}{\partial r}\to 0,T\to T_{\infty }\text{.} \end{array}$$

The heat flux that radiates may be represented as follows when the Rosseland approximation for radiation is used, which is relevant for optically thick media according to Ref. [27]:

(6) $$q_{r}=-\frac{4\sigma^{*}}{3k^{*}}\frac{\partial T^{4}}{\partial r}=-\frac{16\sigma^{*}}{3k^{*}}T_{\infty }^{3}\frac{\partial T}{\partial r}\text{,}$$

Non-dimensional temperature can be given as:

(7) $$\theta \left(\eta \right)=\frac{T-T_{\infty }}{T_{f}-T_{\infty }}\text{,}$$

with

(8) $$T=T_{\infty }\left[\left(\theta_{w}-1\right)\theta +1\right]\text{,}$$

and $\theta_{w}=\frac{T_{f}}{T_{\infty }}\text{.}$.

To get a similarity solution for the flow equations, the dimensionless variables are defined as [27]:

(9) $$\eta =\sqrt{\frac{a}{\nu_{f}}}r,p=\rho_{f}s^{2}a^{2}P\left(\eta \right),u=asf^{\prime }\left(\eta \right),v=-\frac{R}{R+r}\sqrt{a\nu_{f}}f\left(\eta \right)\text{.}$$

By utilizing equation (9), the equation for continuity is automatically fulfilled, using Table 1 and equations (2), (4) lead to:

(10) $$\frac{\partial P}{\partial \eta }=\frac{\rho_{tf}}{\rho_{f}}\frac{{\left(f^{\prime }\right)}^{2}}{K+\eta }\text{,}$$

(11) $$\frac{\rho_{f}}{\rho_{\text{tf}}}\frac{2\text{KP}}{K+\eta }=\frac{\nu_{\text{tf}}}{\nu_{f}}\left(1+\frac{1}{\beta_{1}}\right)\left(f^{\prime \prime \prime }+\frac{f^{\prime \prime }}{K+\eta }‐\frac{f^{\prime }}{{\left(K+\eta \right)}^{2}}\right)‐\frac{K{\left(f^{\prime }\right)}^{2}}{K+\eta }+\frac{\text{Kf}f^{\prime \prime }}{K+\eta }+\frac{\text{Kf}f^{\prime }}{{\left(K+\eta \right)}^{2}}‐\frac{\sigma_{\text{tf}}\rho_{f}}{\sigma_{f}\rho_{\text{tf}}}Mf^{\prime }\text{,}$$

(12) $$\frac{1}{\mathrm{Pr}}\frac{\nu_{\text{tf}}}{\nu_{f}}\left\{\left(K+\eta \right)\left(\frac{K_{f}}{K_{\text{tf}}}\text{Rd}{\left[\left(\theta_{w}‐1\right)\theta +1\right]}^{3}+1\right)\theta^{\prime \prime }+\left(\frac{K_{f}}{K_{\text{tf}}}\text{Rd}{\left[\left(\theta_{w}‐1\right)\theta +1\right]}^{3}+1\right)\theta^{\prime }\right\}+\frac{K}{K+\eta }f\theta^{\prime }=0\text{,}$$

Where

$$\begin{array}{l} K=R\sqrt{\frac{a}{\nu_{f}}},\mathrm{Pr}=\frac{\nu_{f}}{\alpha_{f}},M=\frac{\sigma_{f}}{\rho_{f}a}B_{0}^{2},Rd=\frac{16\sigma^{*}T_{\infty }^{3}}{3K_{f}k^{*}},L_{1}=\sqrt{\frac{a}{\nu_{f}}}L,Bi=\frac{h_{f}}{K_{f}}\sqrt{\frac{\nu_{f}}{a}}\text{,}\\ A_{1}=\frac{\nu_{tf}}{\nu_{f}},A_{2}=\frac{\rho_{tf}}{\rho_{f}},A_{3}=\frac{\sigma_{tf}}{\sigma_{f}},A_{4}=\frac{K_{tf}}{K_{f}},A_{5}=\frac{\mu_{tf}}{\mu_{f}}\text{.} \end{array}$$

**Table 1:** **Thermophysical properties oftrihybrid nanofluid used in this study** [29,30].

Properties	Trihybrid nanofluid
Dynamic Viscosity	$\mu_{tf}=\frac{\mu_{f}}{{\left(1-\varphi_{1}\right)}^{2.5}{\left(1-\varphi_{2}\right)}^{2.5}{\left(1-\varphi_{3}\right)}^{2.5}}$
Density	$\rho_{tf}=\left(1-\varphi_{3}\right)\left\{\left(1-\varphi_{2}\right)\left[\rho_{f}\left(1-\varphi_{1}\right)+\rho_{1}\varphi_{1}\right]+\rho_{2}\varphi_{2}\right\}+\rho_{3}\varphi_{3}$
Electrical conductivity	$\begin{array}{l} \sigma_{nf}=\sigma_{f}\left(\frac{\sigma_{1}+2\sigma_{f}-2\varphi_{1}\left(\sigma_{f}-\sigma_{1}\right)}{\sigma_{1}+2\sigma_{f}+\varphi_{1}\left(\sigma_{f}-\sigma_{1}\right)}\right)\\ \sigma_{hf}=\sigma_{nf}\left(\frac{\sigma_{2}+2\sigma_{nf}-2\varphi_{2}\left(\sigma_{nf}-\sigma_{2}\right)}{\sigma_{2}+2\sigma_{nf}+\varphi_{2}\left(\sigma_{nf}-\sigma_{2}\right)}\right)\\ \sigma_{tf}=\sigma_{hf}\left(\frac{\sigma_{3}+2\sigma_{hf}-2\varphi_{3}\left(\sigma_{hf}-\sigma_{3}\right)}{\sigma_{3}+2\sigma_{hf}+\varphi_{3}\left(\sigma_{hf}-\sigma_{3}\right)}\right) \end{array}$
Thermal conductivity	$\begin{array}{l} K_{nf}=K_{f}\left(\frac{K_{1}+2K_{f}-2\varphi_{1}\left(K_{f}-K_{1}\right)}{K_{1}+2K_{f}+\varphi_{1}\left(K_{f}-K_{1}\right)}\right)\\ K_{hf}=K_{nf}\left(\frac{K_{2}+2K_{nf}-2\varphi_{2}\left(K_{nf}-K_{2}\right)}{K_{2}+2K_{nf}+\varphi_{2}\left(K_{nf}-K_{2}\right)}\right)\\ K_{tf}=K_{hf}\left(\frac{K_{3}+2K_{hf}-2\varphi_{3}\left(K_{hf}-K_{3}\right)}{K_{3}+2K_{hf}+\varphi_{3}\left(K_{hf}-K_{3}\right)}\right) \end{array}$
Heat capacity	$\begin{array}{l} {\left(\rho C_{p}\right)}_{\text{tf}}=\left(1‐\varphi_{3}\right)\left\{\left(1‐\varphi_{2}\right)\left[{\left(\rho C_{p}\right)}_{f}\left(1‐\varphi_{1}\right)+{\left(\rho C_{p}\right)}_{1}\varphi_{1}\right]+{\left(\rho C_{p}\right)}_{2}\varphi_{2}\right\}+{\left(\rho C_{p}\right)}_{3}\varphi_{3} \end{array}$

The associated boundary circumstances becomes:

(13) $$\begin{array}{c} f\left(0\right)=0,f^{\prime }\left(0\right)=1+L_{1}\left(f^{\prime \prime }\left(0\right)-\frac{f^{\prime }\left(0\right)}{K}\right),\theta^{\prime }\left(0\right)=-\frac{Bi}{A_{4}}\left[1-\theta \left(0\right)\right]\text{,}\\ f^{\prime }\left(\infty \right)\to 0,f^{\prime \prime }\left(\infty \right)\to 0,\theta \left(\infty \right)\to 0\text{.} \end{array}$$

Removing the pressure among equations (10), (11) gives:

(14) $$A_{1}\left(1+\frac{1}{\beta_{1}}\right)\left({f^{\prime }}^{v}+\frac{2f^{\prime \prime \prime }}{K+\eta }‐\frac{f^{\prime \prime }}{K+\eta }\right)+\frac{K\left(f\;f^{\prime \prime \prime }‐f^{\prime }\;f^{\prime \prime }\right)}{K+\eta }+\frac{K\left(f\;f^{\prime \prime }‐{\left(f^{\prime }\right)}^{2}\right)}{{\left(K+\eta \right)}^{2}}‐\frac{\text{Kf}\;f^{\prime }}{{\left(K+\eta \right)}^{3}}‐\frac{A_{3}}{A_{2}}M\left(\frac{f^{\prime }}{K+\eta }+f^{\prime \prime }\right)=0.$$

After the fluid velocity profile is obtained, equation (11) can be used to calculate the pressure.

The important physical parameters are the skin friction and the rate of heat exchange in the s-direction, given as [27]:

(15) $$C_{f}=\frac{\tau_{rs}}{\rho_{f}U_{w}^{2}}\text{,}$$

(16) $$Nu_{s}=\frac{sq_{w}}{K_{f}\left(T_{f}-T_{\infty }\right)}\text{,}$$

$$\text{Here}\;\tau_{rs}=-\mu_{tf}\left(1+\frac{1}{\beta_{1}}\right){\left(\frac{\partial u}{\partial r}-\frac{u}{R+r}\right)|}_{r=0},q_{w}=-{K_{tf}\frac{\partial T}{\partial r}+q_{r}|}_{r=0}\text{.}$$

By utilizing equation (9), equations (15), (16) can be expressed as:

(17) Res12Cf=A5(1β1+1)(f′(0)K−f″(0)),

(18) Res−12Nus=−A4{RdA4[(θw−1)θ+1]3+1}θ′(0).

## 3 Numerical procedure

**Fig. (2):**
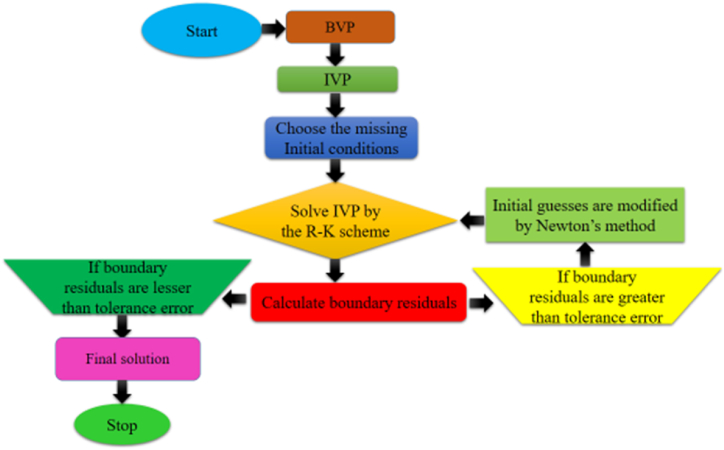
Flow chart for numerical procedure.

Dimensionless equations (12), (13), (14) are employed in constructing a non-linear boundary value problem. The combination of the shooting technique and the R–K technique is utilized to solve this set of non-linear ordinary differential equations, with a boundary value solver implemented through the MATLAB program. Fig. 2 illustrates the flow chart outlining the numerical operations executed by the MATLAB-based boundary value problem. Before coding, certain assumptions must be considered as.

(19) $$f=f\left(1\right),f^{\prime }=f\left(2\right),f^{\prime \prime }=f\left(3\right),f^{\prime \prime \prime }=f\left(4\right),\theta =f\left(5\right),\theta^{\prime }=f\left(6\right)\text{.}$$

Using the assumptions stated in (19), construct the following system of first-order ordinary differential equations based on equations (12), (14), and along the boundary conditions (13) are

(20) $$\begin{array}{c} f^{\prime }\left(1\right)=f\left(2\right)\text{,}\\ f^{\prime }\left(2\right)=f\left(3\right)\text{,}\\ f^{\prime }\left(3\right)=f\left(4\right)\text{,}\\ f^{\prime }\left(4\right)=-\frac{1}{A_{1}\left(\frac{1}{\beta_{1}}+1\right)}\{\frac{Kf\left(1\right)f\left(2\right)}{{\left(K+\eta \right)}^{3}}-\frac{K\left[f\left(1\right)f\left(4\right)-f\left(2\right)f\left(3\right)\right]}{\left(K+\eta \right)}-\frac{K\left[f\left(1\right)f\left(3\right)-{\left(f\left(2\right)\right)}^{2}\right]}{\left(K+\eta \right)}+\frac{A_{3}}{A_{2}}M\left(f\left(3\right)+\frac{f\left(2\right)}{\left(K+\eta \right)}\right)\}+\frac{f\left(3\right)}{{\left(K+\eta \right)}^{2}}-\frac{2f\left(4\right)}{\left(K+\eta \right)}\text{,}\\ f^{\prime }\left(5\right)=f\left(6\right)\text{,}\\ f^{\prime }\left(6\right)=-\frac{1}{\left(K+\eta \right)}\;\left\{\frac{\mathrm{Pr}\;Kf\left(1\right)}{A_{1}\left(K+\eta \right)\left\{\frac{Rd}{A_{4}}{\left[1+\left(\theta_{w}-1\right)f\left(5\right)\right]}^{3}+1\right\}}+1\right\}f\left(6\right)\text{,} \end{array}\}$$

(21) $$\begin{array}{c} fa\left(1\right)=0,fa\left(2\right)=1+L_{1}\left(fa\left(3\right)-\frac{fa\left(2\right)}{K}\right),fa\left(6\right)=-Bi\frac{1}{A_{4}}\left[1-fa\left(5\right)\right]\text{,}\\ fb\left(2\right)=0,fb\left(3\right)=0,fb\left(5\right)=0\text{.} \end{array}$$

After converting the system mentioned above to MATLAB code and setting the error tolerance to 10^−6^, execute it to acquire the results as in graphs and tables.

## 4 Discussion

**Fig. (3):**
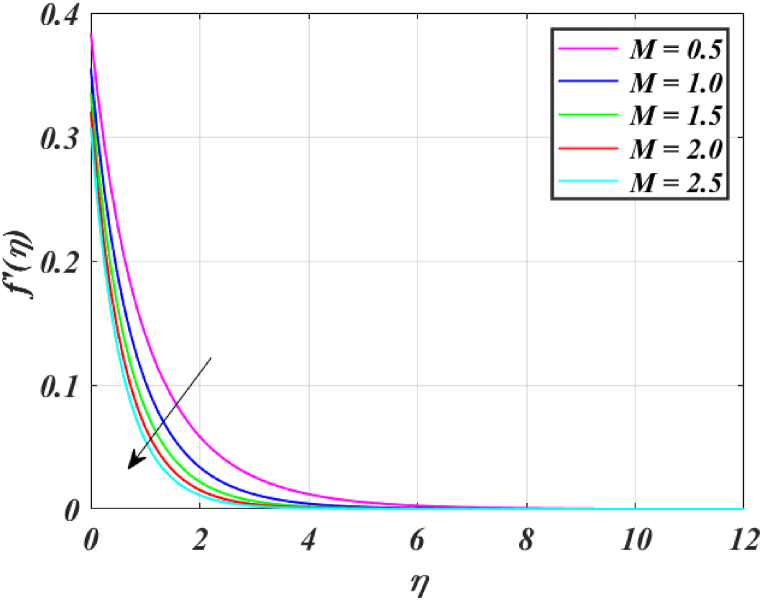
$f^{\prime }\left(\eta \right)$ for different *M*.

**Fig. (4):**
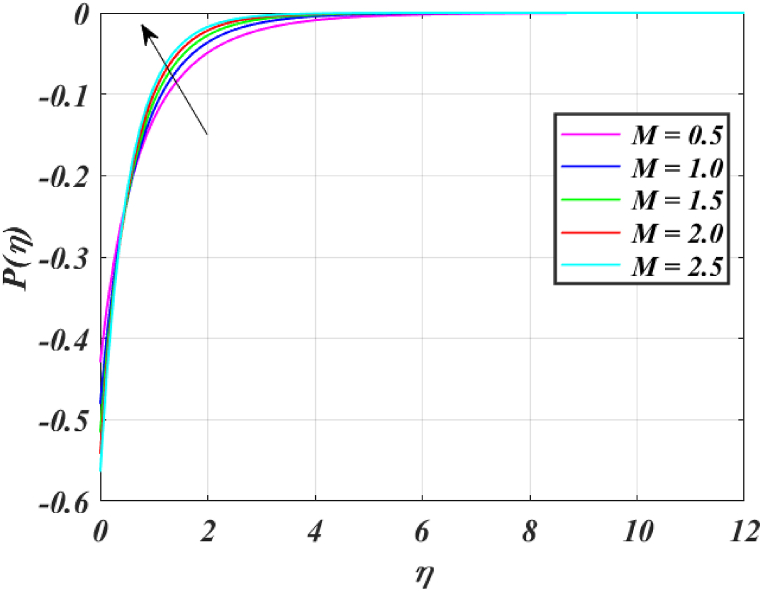
P(η) for different *M*.

**Fig. (5):**
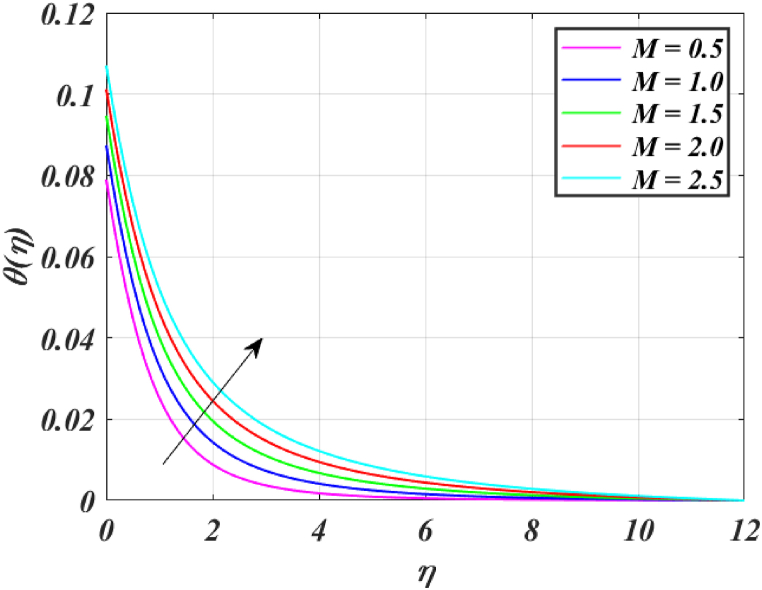
θ(η) for different *M*.

**Fig. (6):**
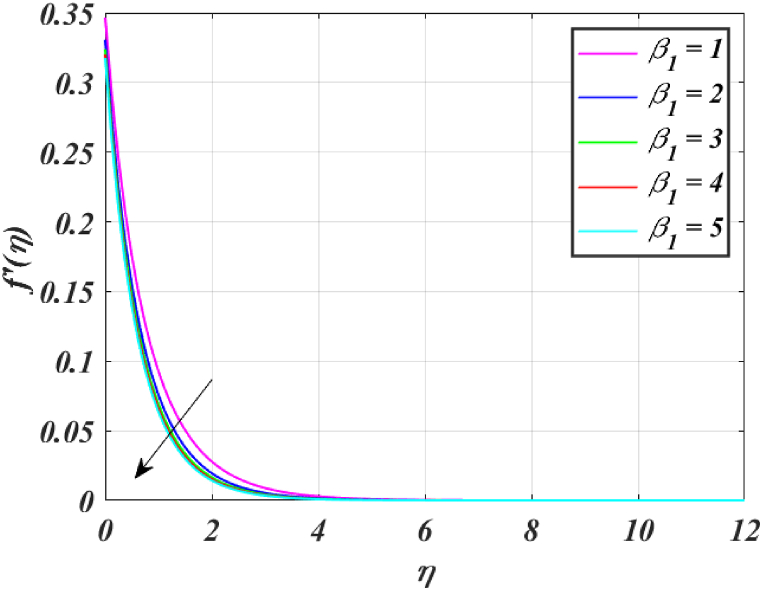
$f^{\prime }\left(\eta \right)$ for different $\beta_{1}$.

**Fig. (7):**
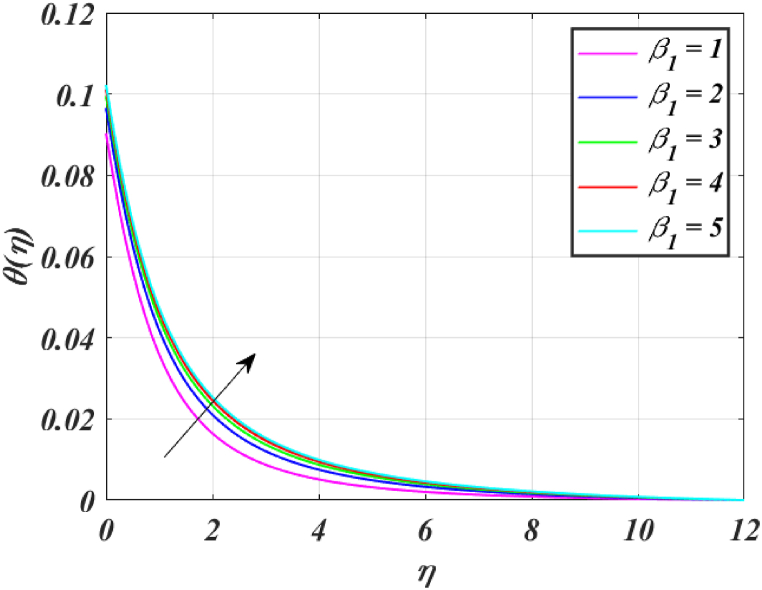
θ(η) for different $\beta_{1}$.

Several control factors identified in this study have been altered in this segment to investigate their impact on the momentum profile, skin friction coefficient, pressure, thermal profile and rate of heat transfer of the (*GO-SWCNTs-MWCNTs/Blood*) trihybrid nanofluid. Tables, and graphs have been utilized to present the findings in the current study. For finding the results fixing the key parameter values $M=2,K=1.5,\beta_{1}=4,L_{1}=0.9,\mathrm{Pr}=24,Rd=3,\theta_{w}=1.5\text{,}$ and Bi=0.1 and using Table 2 helps us to understand the problem's physics. Fig. 3 clearly shows that raising *M* reduces the velocity of fluid. This is because it has a magnetic field outside acts as a retarding effect on the fluid. It is also found that the velocity of the liquid falls significantly with the existence of tiny particles due to nanoparticles producing friction in the liquid. Fig. 4 shows that the distribution's magnitude of pressure decreases initially and gradually rises as *M* rises. Fig. 5 shows that increasing *M* causes the fluid's temperature to rise. The actual cause for this phenomenon is that the action of *M* is to drop the fluid movement and the force supplied to the movement, causing a rise in the fluid's temperature. Fig. 6 clearly shows that a rise in $\beta_{1}$ outcomes in a deduction in fluid velocity. This phenomenon may be resulting from the reality that a rise in $\beta_{1}$ causes an increase in plastic dynamic, which in turn produces an opposing force to the fluid's flow and a reduction in fluid velocity. Additionally, when $\beta_{1}$ grows, the thickness of the momentum boundary layer reduces because the yield stress reduces because of an increase $\beta_{1}$, deduce the thickness of the velocity boundary layer. Fig. 7 shows that increasing $\beta_{1}$ leads to increased temperature and thickness of the thermal boundary layer. The augmentation of the thermal boundary layer is attributable to rises in the elasticity stress parameter.

**Table 2:** Thermophysical values of base fluid and nanoparticles used in this study are.

Physical parameters	Blood [15]	GO [31]	SWCNTs [15]	MWCNTs [15]
$\rho \left(kg/m^{3}\right)$	1053	1115	8933	8900
k(W/mK)	0.492	2430	401	90.7
Cp(J/kgK)	3594	0.253	385	444
σ (s/m)	0.8	6.30 × 10^7^	10^6^–10^7^	1.9 × 10^−4^
μ(mPa/s)	2.78	_	_	_
Pr	24	_	_	_

**Fig. (8):**
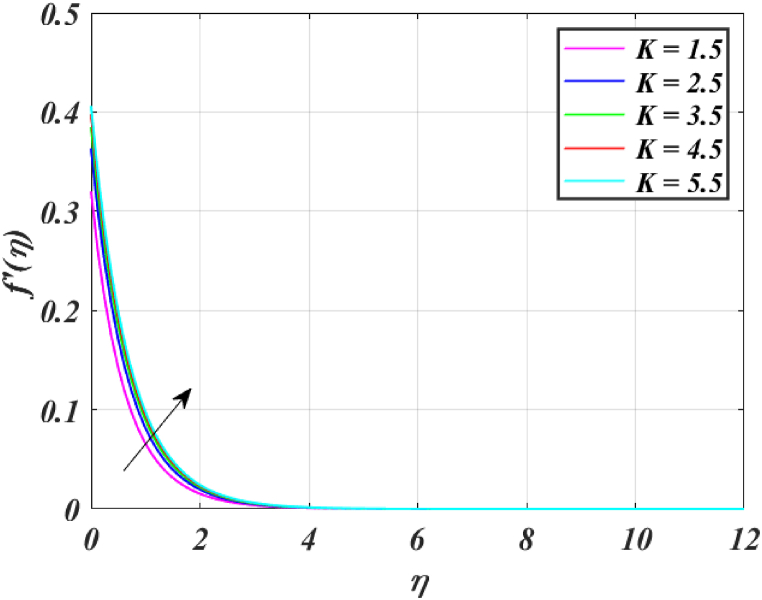
$f^{\prime }\left(\eta \right)$ for different *K*.

**Fig. (9):**
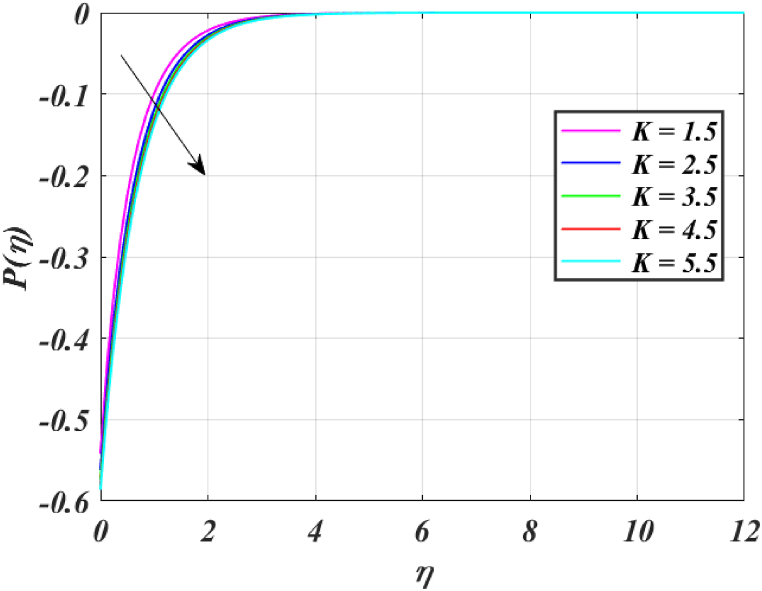
P(η) for different *K*.

**Fig. (10):**
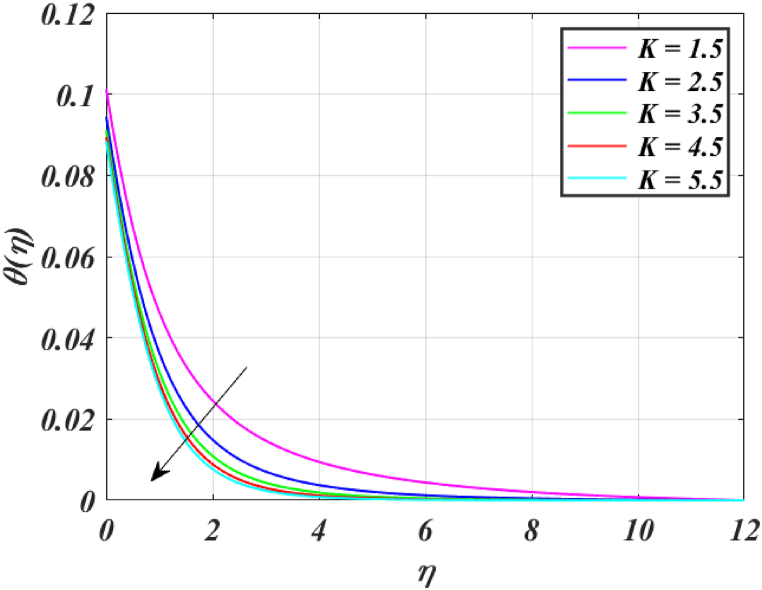
θ(η) for different *K*.

**Fig. (11):**
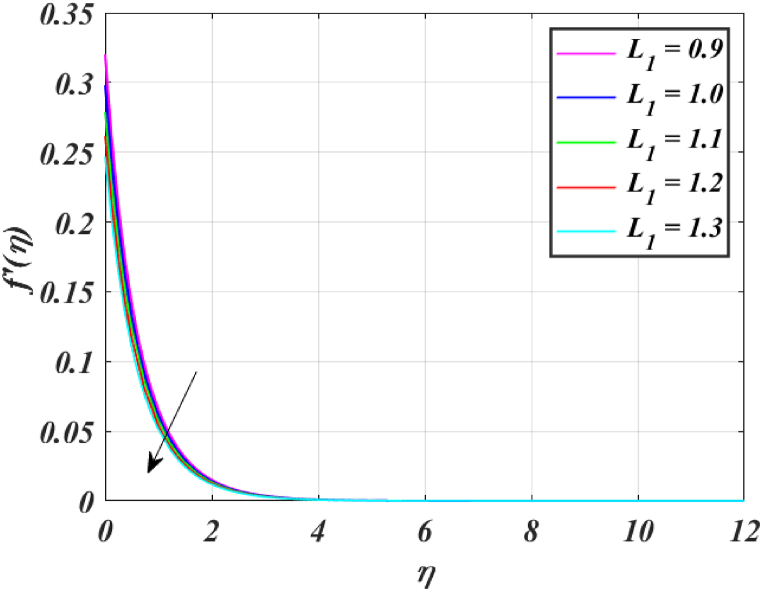
$f^{\prime }\left(\eta \right)$ for different $L_{1}$.

**Fig. (12):**
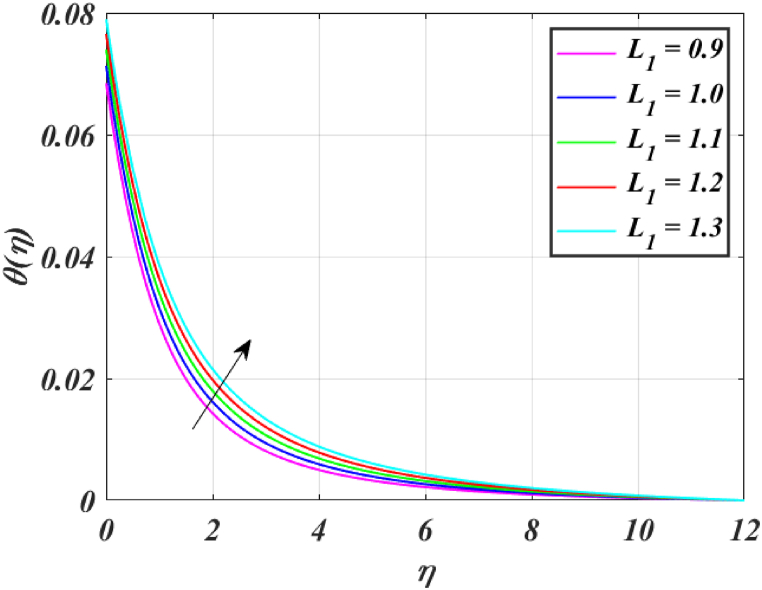
θ(η) for different $L_{1}$.

**Fig. (13):**
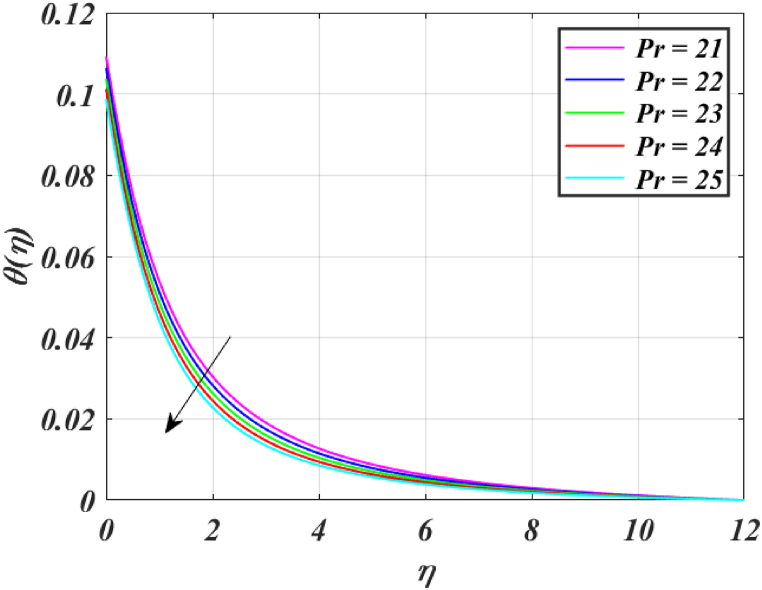
θ(η) for different *Pr*.

**Fig. (14):**
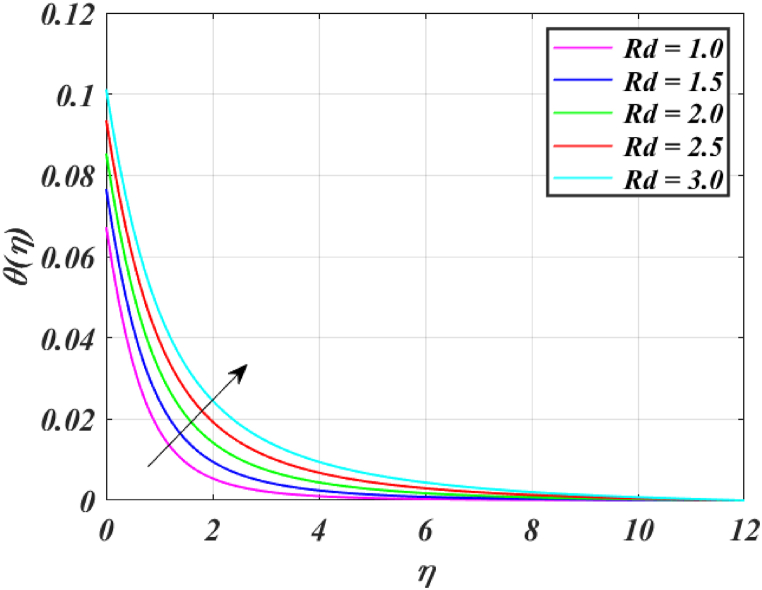
θ(η) for different *Rd*.

**Fig. (15):**
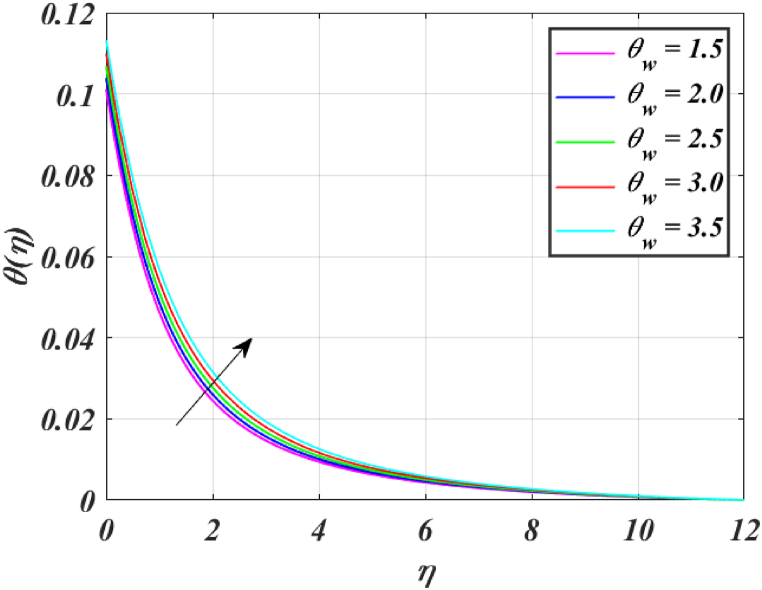
θ(η) for different $\theta_{w}$.

**Fig. (16):**
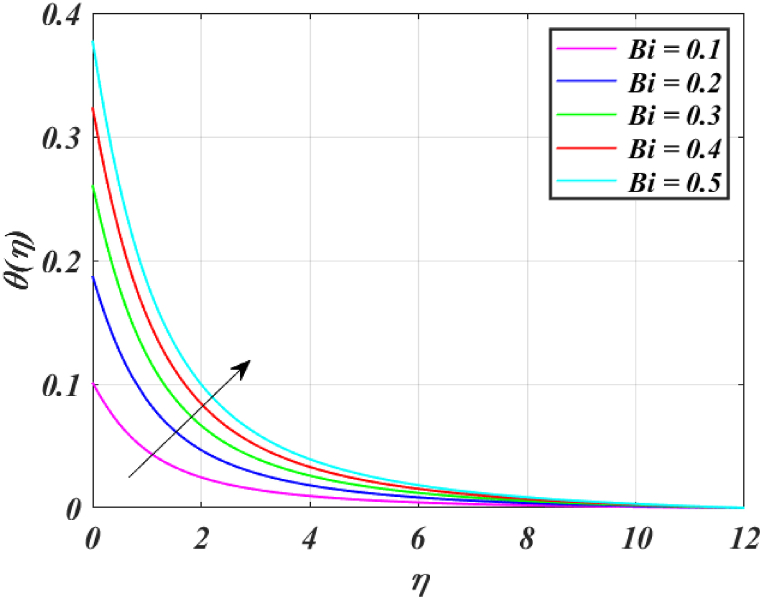
θ(η) for different *Bi*.

Fig. 8 clearly shows that velocity increases with increasing *K*. By increasing the *K*, the viscous force is minimized. Consequently, resistive force reduces, improving the velocity field. Fig. 9 indicates the magnitude of the pressure distribution falls as *K* rises. Fig. 10 shows that fluid temperature decreases as *K* rises. The surface is clearly flat for a huge *K*. In this scenario, the distance between nanoparticles grows as the curved path reduces. It drops the temperature of the fluid. Increasing the value $L_{1}$ reduces the fluid's velocity, as seen in Fig. 11. The drop in fluid velocity at the sheet can be seen by the fact that the flow of fluid is influenced by sheet stretching due to rise in $L_{1}$. Fig. 12 indicates clearly that raising the value of $L_{1}$ results in an increment in both the thermal and the temperature boundary layer. Fig. 13 illustrates that for raised values of *Pr*, the heat of the fluid reduced due to slower thermal diffusion. Furthermore, it is noticed that the thickness of the temperature boundary layer reduces as Pr grows. Fig. 14 shows that raising the value of *Rd* causes fluid's temperature to increase. The actual meaning of this pattern is that increment in the values of *Rd* improves the conduction impacts, resulting in an increment in temperature at all points far from the sheet. As a result, a greater surface heat flux is associated with an increased value of *Rd*. Fig. 15 demonstrates that an incrementing the values of $\theta_{w}$ tends to a rise in the fluid's temperature. The thermal boundary layer thickness also rises with a rise in $\theta_{w}$. Fig. 16 shows that increasing *Bi* resulted in an improvement in the thermal field. *Bi* refers to the fraction of conduction within an item to convection on its surface. Greater *Bi* increases heat transmission by convection, reducing thermal resistance and increasing fluid temperature.

**Fig. (17):**
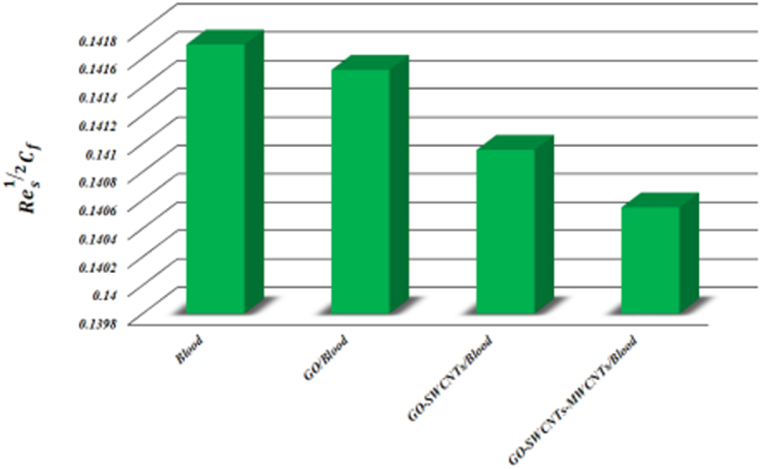
Comparison of *Blood, GO/Blood, GO-SWCNTs/Blood,* and *GO-SWCNTs-MWCNTs/Blood* for Res12Cf.

**Fig. (18):**
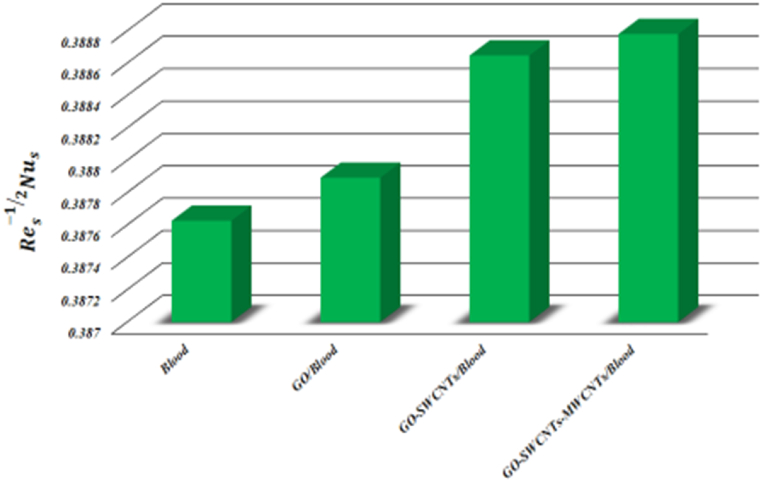
Comparison of *Blood, GO/Blood, GO-SWCNTs/Blood*, and *GO-SWCNTs-MWCNTs/Blood* for Res−12Nus.

Table 3 shows the impact of *M,* $\beta_{1}$, *K*, $L_{1}$, $\theta_{w}$, and *Bi* on the Nusselt number and skin friction coefficient. As $\beta_{1}$ grows, both the heat transfer rate and skin friction coefficient are diminished due to the yield stress reduces because of an increase $\beta_{1}$. As the L value grows, the coefficient of skin friction and heat transmission rate both drop down. Skin friction coefficient drops and Nusselt number rises as *K* grows, indicating a larger rate of heat transmission over curved surface relative to flat surface (*K → ∞*). Raising the values of *M* improves the rate of heat and friction coefficient flow. By increasing the values of $\theta_{w}$ and *Bi* grows the heat transfer rate and reduces the coefficient of skin friction. Keeping other parameters constant, a comparison of *Blood*, *GO/Blood*, *GO-SWCNTs/Blood*, and *GO-SWCNTs-MWCNTs/Blood* is done for both skin friction coefficient and Nusselt number. Fig. 17 depicts the impact of surface drag force. For all fluids, the connection between nanoparticle concentration and Res12Cf is straightforward. Trihybrid nanofluid has lower Res12Cf values than hybrid nanofluid, nanofluid, and base fluid. Fig. 18 depicts the effect of the heat transfer rate. Trihybrid nanofluid has higher Res−12Nus values compared to hybrid nanofluid, nanofluid, and base fluid.

**Table 3:** Keeping $Rd=3,\mathrm{Pr}=24,\varphi_{1}=\varphi_{2}=\varphi_{3}=0.01$ then skin friction coefficient and Nusselt number for different parameters.

*M*	$\beta_{1}$	*K*	$L_{1}$	$\theta_{w}$	*Bi*	Res12Cf	Res−12Nus
0.1						0.860568	0.375842
0.2						0.881498	0.375856
0.3						0.897957	0.375864
	1.5					1.324581	0.375837
	2.0					1.202859	0.375821
	2.5					1.129322	0.375807
		5				0.895077	0.375863
		10				0.862671	0.375871
		20				0.845746	0.375873
			0.6			1.320002	0.375869
			0.7			1.201155	0.375849
			0.8			1.102089	0.375819
				1.5		1.018216	0.375781
				2.0		1.018216	0.420283
				2.5		1.018216	0.471992
					0.1	1.018216	0.375781
					0.2	1.018216	0.745750
					0.3	1.018216	1.100374

## 5 Validation

The code was validated and the accuracy of the results was verified by comparing them with previously published research by Abbas et al., [6]. Table 4 displays Res12Cf for many values of *L*_*1*_ when $K=1000,M=0,\theta_{w}=0,Bi=0,and\;\beta_{1}\to \infty$, indicating that the results of this investigation are consistent with those that have been published before.

**Table 4:** A comparison of results for several choices of *L*_*1*_ when $K=1000,M=0,\theta_{w}=0,Bi=0,and\;\beta_{1}\to \infty$.

$L_{1}$	Abbas et al. [6],	Present results
0.0	1.0000	1.0007
0.1	0.8720	0.8726
0.2	0.7763	0.7768
0.3	0.7015	0.7019
0.5	0.5911	0.5915
1.0	0.4301	0.4304
2.0	0.2839	0.2841
3.0	0.2140	0.2142
5.0	0.1448	0.1450
10	0.0812	0.0814

## 6 Key findings

The primary findings are listed below**:**

➢ Reduction arises in the velocity profile as raising the magnetic parameter.
➢ Increasing the curvature parameter increases velocity.
➢ Larger slip parameters result in a reduction in velocity.
➢ Increasing Casson parameter decreases velocity and opposite trend in temperature.
➢ Temperature increases as raising the magnetic parameter.
➢ ‵As *Bi* grows, the thermal field grows more effective.
➢ Trihybrid nanofluid has lesser values for coefficient of skin friction compared to hybrid nanofluid, nanofluid, and base fluid.
➢ Trihybrid nanofluid has larger values for heat transfer rate compared to hybrid nanofluid, nanofluid, and base fluid.

## Future scope

The researchers can involve effects like porous medium, stagnation point, heat source/sink etc., and involve concentration equation with chemical reaction and so on.

## Data availability

The data that has been used is simulated from this study.

## CRediT authorship contribution statement

**Bhargavi N:** Writing – original draft, Software, Methodology, Investigation, Formal analysis, Conceptualization. **Poornima T:** Writing – review & editing, Validation, Supervision, Methodology, Investigation, Formal analysis, Conceptualization.

## Declaration of competing interest

The authors declare that they have no known competing financial interests or personal relationships that could have appeared to influence the work reported in this paper.
